# Impact of High-Temperature Feeds on Gut Microbiota and MAFLD

**DOI:** 10.4014/jmb.2405.05023

**Published:** 2024-07-31

**Authors:** Lijun Xue, Kaimin Li, Yanfei Jia, Dongxue Yao, Xuexing Guo, Shuhong Zhang

**Affiliations:** 1 Digestive Department 2, Jinan Central Hospital Affiliated to Shandong First Medical University, Jinan 250013, P.R. China; 2 Department of Gastroenterology, China-Japan Friendship Hospital, Beijing 100029, P.R. China; 3 Research Center of Basic Medicine, Jinan Central Hospital, Jinan 250013, P.R. China

**Keywords:** Metabolically associated fatty liver disease, high-temperature processed feed, sprague-dawley rats, fecal flora characteristics, pathogenesis of non-obese metabolically associated fatty liver disease

## Abstract

The purpose of this study is to investigate the effects of non-obese MAFLD on the gut microbiota and metabolic pathways caused by high-temperature processed meals. It was decided to divide the eighteen male Sprague-Dawley rats into three groups: the control group, the dry-fried soybeans (DFS) group, and the high-fat diet (HFD) group. Following the passage of twelve weeks, a series of physical, biochemical, histological, and microbiological examinations were carried out. There were distinct pathological abnormalities brought about by each diet. The DFS diet was found to cause the development of fatty liver and to demonstrate strong relationships between components of the gut microbiota, such as *Akkermansia* and *Mucispirillum*, and indices of liver health. Diet-induced changes in the gut microbiome have a significant impact on liver pathology in non-obese patients with metabolically altered liver disease (MAFLD), which suggests that dietary interventions that target gut microbiota could be used to manage or prevent the illness.

## Introduction

Non-alcoholic fatty liver disease (NAFLD) is widely recognized as a liver disorder, previously considered solely associated with obesity [1]. However, it is now understood to be related to metabolic dysfunction [2-5]. Recently, some experts have proposed renaming it to metabolically associated fatty liver disease (MAFLD) to more accurately reflect its metabolic nature [6-9]. MAFLD has emerged as a significant health challenge globally, with an increasing prevalence [10-13].

The liver and intestines are closely linked in anatomical structure and physiological function, establishing a tight internal connection through various pathways [14-16]. Mechanisms such as the portal venous blood circulation, the hepatoenteral lymphatic system, and the bile acid cycle of the biliary system facilitate a close interaction between the intestines and the liver [17]. Usually, the liver efficiently eliminates toxins, bacteria, and fungi originating from the intestines, maintaining overall internal stability [18-20]. However, this coordination can be disrupted during the progression of liver diseases, leading to an imbalance in the interaction between the intestines and the liver [21-23]. The pathogenesis of MAFLD involves multiple factors, among which the "gut-liver axis" theory plays a crucial role [8, 10, 21, 22, 24].

In the pathological state of liver disease, patients often present with gastrointestinal abnormalities [25-27]. These may include gastrointestinal inflammation and dysbiosis, negatively impacting the integrity of the intestinal mucosal barrier [21, 22, 28-30]. Intestinal permeability can be assessed through markers such as zonulin levels and lactulose/mannitol ratio. Diet plays a crucial role in regulating the gut flora and host-microbe interactions [22, 31-34]. Different dietary patterns can significantly affect the composition and function of the gut flora, potentially impacting liver health [35, 36]. For example, high-temperature processed foods can initiate oxidative damage, producing many free radicals that damage bodily tissues [37]. Oxidative damage to the intestinal mucosa may compromise the gut barrier, further promoting liver inflammation and fibrosis [38-42].

In our previous research, we successfully established a rat model of non-obese MAFLD induced by high-temperature processed feed, offering an opportunity to profoundly investigate the pathogenesis of MAFLD [43]. To gain a more comprehensive understanding of the impact of high-temperature processed feed on rats, we further explored changes in fecal metabolites and microbial communities, examining the potential correlation between microbial communities and liver fibrosis and the liver index. This study aims to provide new insights into the pathogenesis of MAFLD, potentially offering essential clinical guidance for future preventive and therapeutic strategies.

## Materials and Methods

### Experimental Animals and Diets

In this study, we utilized eighteen male Sprague-Dawley rats, which, after a 2-week acclimatization period, were randomly divided into three groups of six each. The control group received a standard diet (Sulabio, regular maintenance feed, China), the dry-fried soybeans group (DFS group) was fed a diet comprising 40% base diet and 60% high-temperature DFS, and the high-fat diet group (HFD group) received a diet with 45% of its energy from fat. After week 12, all rats were euthanized under sodium pentobarbital anesthesia, and their blood, liver tissues, and feces were collected. This study adhered strictly to relevant ethical standards and regulations, ensuring animal welfare and the ethical conduct of scientific research. All experimental procedures were approved by the Ethics Committee of Jinan Central Hospital Affiliated to Shandong First Medical University (JNCH2021-142) and conducted by the Declaration of Helsinki. Furthermore, all experimental manipulations were performed under the supervision of professionally trained personnel to minimize animal distress and discomfort.

### Measurement of Body Weight, Liver Index, ALT, AST, TC and TG

In this study, body weight measurements were taken for each of the three groups of rats. Following the conclusion of the experiment, the rats were euthanized, and their livers were promptly extracted. The liver's surface blood and attached tissues were removed before weighing to determine the wet weight.

The liver index (%) was calculated using the formula: (liver weight/body weight) × 100%.

Serum alanine aminotransferase (ALT) levels were assessed using a commercial kit (Qi Ming Biotech, China). For this analysis, 1 g of liver tissue was homogenized in 9 ml of PBS, centrifuged for 20 min, and the supernatant was collected. The absorbance was measured at a wavelength of 450 nm according to the kit's instructions. Similarly, aspartate aminotransferase (AST) levels were determined using a kit from Lai'er Biotech (Hefei, China), where 0.1 g of liver tissue was homogenized in 1 ml of extraction buffer, centrifuged at 3500 g at 4°C for 10 min, and the supernatant was used for the assay. Absorbance was measured at 450 nm as per the provided protocol.

The concentrations of cholesterol (TC) and triglycerides (TG) were measured using a kit from Lai'er Biotech (China), following a similar procedure to that used for AST measurement. Liver tissue (0.1 g) was homogenized in 1 ml of extraction buffer and centrifuged, and the supernatant's absorbance was measured at 450 nm wavelength according to the kit's instructions.

### Liver Tissue Pathology

Building upon previous research [43-45], this study assessed liver tissue pathology indicators. The evaluation followed established protocols for Oil Red O and Masson's trichrome staining. The severity of liver fibrosis was assessed using the METAVIR scoring system. This system categorizes fibrosis into five stages: 0 (no fibrosis), 1 (mild fibrosis), 2 (moderate fibrosis), 3 (severe fibrosis), and 4 (cirrhosis). Each tissue sample was evaluated independently by two pathologists to ensure the accuracy and consistency of the scoring.

### Intestinal Health Assessment

Intestinal health was evaluated by measuring intestinal integrity and permeability. Intestinal permeability was assessed using serum zonulin levels and the lactulose/mannitol test. Serum zonulin levels were measured using an ELISA kit following the manufacturer’s instructions. Blood samples were collected from the rats, and the serum was separated by centrifugation. The serum samples were then subjected to the ELISA procedure to quantify zonulin levels. The lactulose/mannitol test was conducted by administering a solution containing lactulose and mannitol to the rats via oral gavage. Following the administration, urine was collected over a specified period, typically 6 hours. The collected urine samples were analyzed using high-performance liquid chromatography (HPLC) to determine the concentrations of lactulose and mannitol. The lactulose/mannitol ratio was then calculated to determine intestinal permeability, with higher ratios indicating increased permeability.

### Microbial Diversity Sequencing and Analysis

Each rat group collected fecal samples for microbial diversity sequencing on the Illumina MiSeq PE300 platform. Alpha diversity, reflecting microbial community diversity, was analyzed using metrics such as the Shannon index, which considers the proportion of each category. The Ace and Chao indices were used to describe species richness. Based on species annotation results, community composition was analyzed through bar charts, heatmaps, and Venn diagrams, with key bacterial populations identified. Principal component analysis (PCA) was conducted to investigate compositional differences and similarities, and correlation heat maps were utilized to analyze associations between bacterial communities and metabolites.

### Metabolomics Analysis

Metabolic profiling of fecal samples from the eighteen rats at 12 weeks was conducted using liquid chromatography-mass spectrometry (LC-MS). Metabolites were extracted from fecal samples using a liquid-liquid extraction method and dissolved in an appropriate extraction solvent. The extracted metabolites were then centrifuged and filtered to obtain a clean extract for analysis. Correlations between fecal metabolites and microbial communities in the DFS group compared to the control and HFD groups were examined, with red and blue indicating positive and negative correlations, respectively. Statistical analyses were based on our prior research [43].

### Statistical Analysis

Various statistical methods were employed to analyze and interpret the data. Continuous variables such as body weight and liver index were compared using analysis of variance (ANOVA), conducted with GraphPad Prism 8.0 software (USA). Microbial diversity within samples was assessed using Shannon, Chao, and Ace indices to evaluate species diversity and richness. Beta diversity was evaluated through PCA to reveal differences in community composition.

## Results

### Impact of High-Temperature Processed Feed on Rat Body Weight and Liver Index in Non-Obese MAFLD

**Fig. 1:**
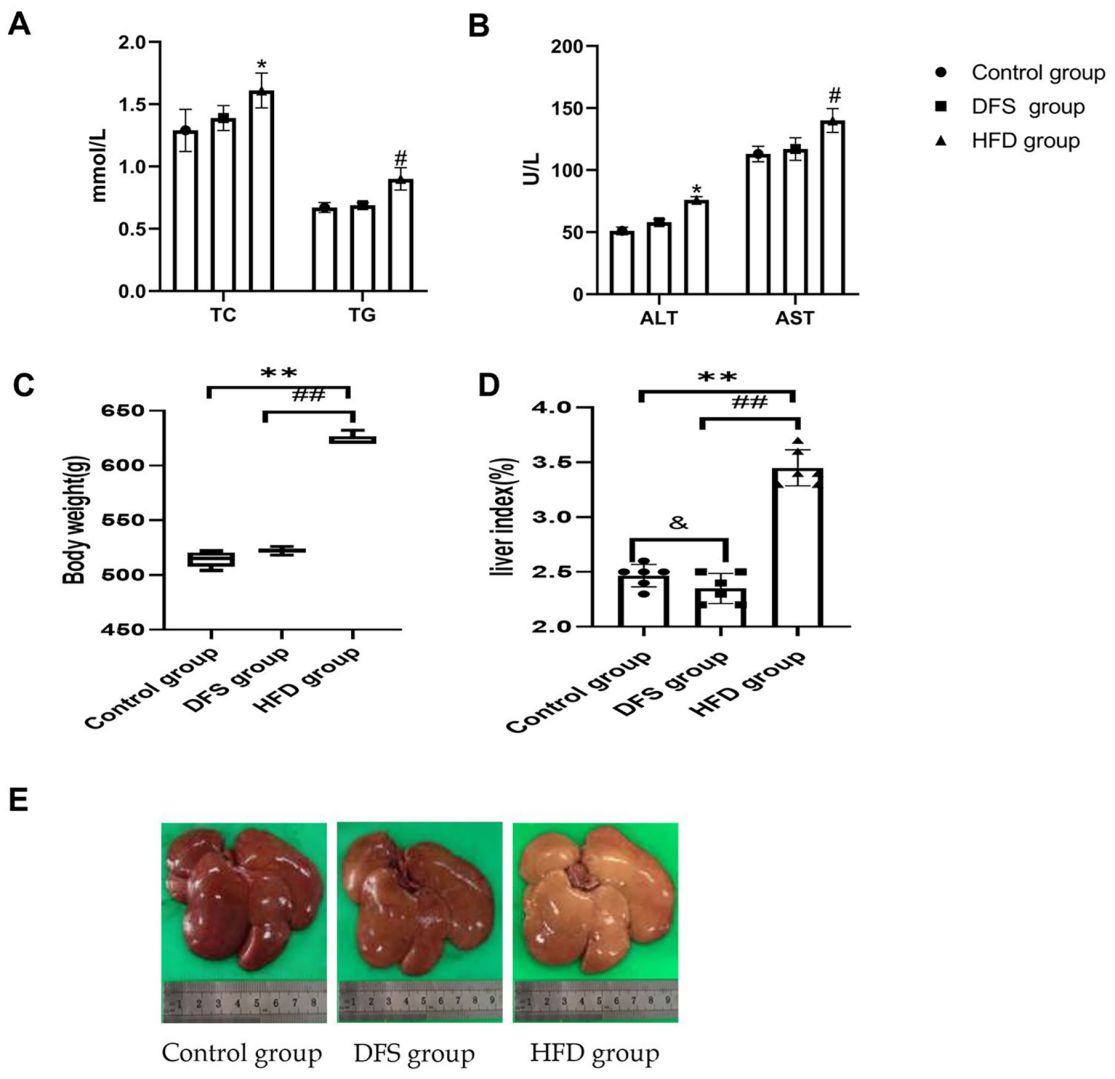
Effects of high-temperature processed feed on rat body weight and liver index. (**A**) Serum lipid levels [*p < 0.05, TC: comparison between HFD group and the other two groups; #*p* < 0.05, TG: comparison between HFD group and the other two groups]; (**B**) Serum enzyme levels [*p < 0.05, ALT: HFD group compared to the other two groups; #*p* < 0.05, AST: HFD group compared to the other two groups]; (**C**) Body weight [**p < 0.01, ##*p* < 0.01, HFD group compared to the other two groups]; (**D**) Liver index [**p < 0.01, ##*p* < 0.01, HFD group compared to the other two groups; & *p* < 0.05, control group compared to DFS group]; (**E**) Liver morphology across the three groups, with all experiments replicated six times.

**Fig. 2:**
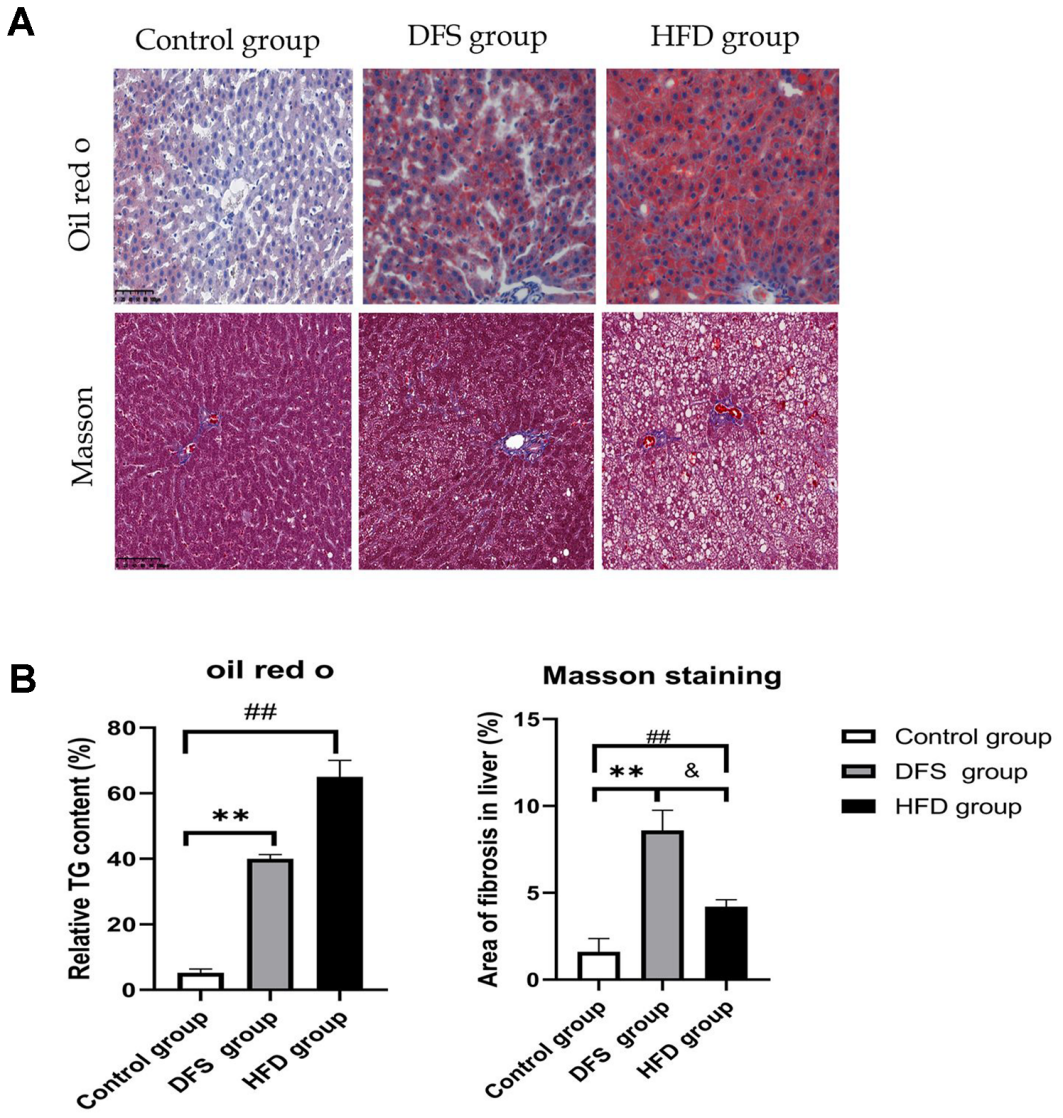
Impact of high-temperature processed feed on rat liver pathology. (**A**) Hepatic fat degeneration and fibrosis in rats at week 12. Scale bar = 100 μm; (**B**) Semiquantitative analysis of the relative percentage of TG content and fibrosis (Oil Red O staining: **p < 0.01, comparison between DFS group and control group, ##*p* < 0.01, comparison between HFD group and control group; Masson's trichrome staining: **p < 0.01, ##*p* < 0.01, comparison of DFS and HFD groups with control group; & *p* < 0.05, comparison between DFS and HFD groups), with all experiments replicated six times.

This study investigated the effects of high-temperature processed feed on body weight and liver index in a rat model of non-obese MAFLD. At week 12, no significant differences were observed in serological markers or body weight between the DFS group and the control group (Fig. 1A-1C) (*p* > 0.05). Compared to the DFS and control groups, the HFD group exhibited a significant increase in liver index and body weight (*p* < 0.01). The liver fat in the DFS group primarily manifested as vesicular steatosis, whereas in the HFD group, it predominantly appeared as bullous steatosis. The liver index in the DFS group was significantly lower than that in both the HFD and control groups (*p* < 0.05, *p* < 0.01) (Fig. 1D). The DFS group showed significant increases in TG content and fibrosis area compared to the control group (*p* < 0.01), demonstrating the impact of DFS on liver pathology (*p* < 0.01) (Fig. 1E). The fibrosis area in the DFS group was significantly greater than in both the control and HFD groups (*p* < 0.01, *p* < 0.05) (Fig. 2A and 2B, Table 1).

To evaluate the impact of high-temperature processed feed on intestinal health, intestinal permeability and integrity were measured using serum zonulin levels and the lactulose/mannitol test. The results indicated that both the DFS and HFD groups exhibited significantly higher serum zonulin levels and lactulose/mannitol ratios compared to the control group, indicating increased intestinal permeability. Specifically, the DFS group showed moderate increases in these indicators, while the HFD group demonstrated the highest levels, suggesting more severe impairment of intestinal integrity (Table 2).

These results indicate that an HFD diet significantly increases liver index, body weight, and manifestations of bullous steatosis, alongside marked increases in TG content and fibrosis area.

### Impact of High-Temperature Processed Feed on Rat Gut Microbial Diversity

**Fig. 3:**
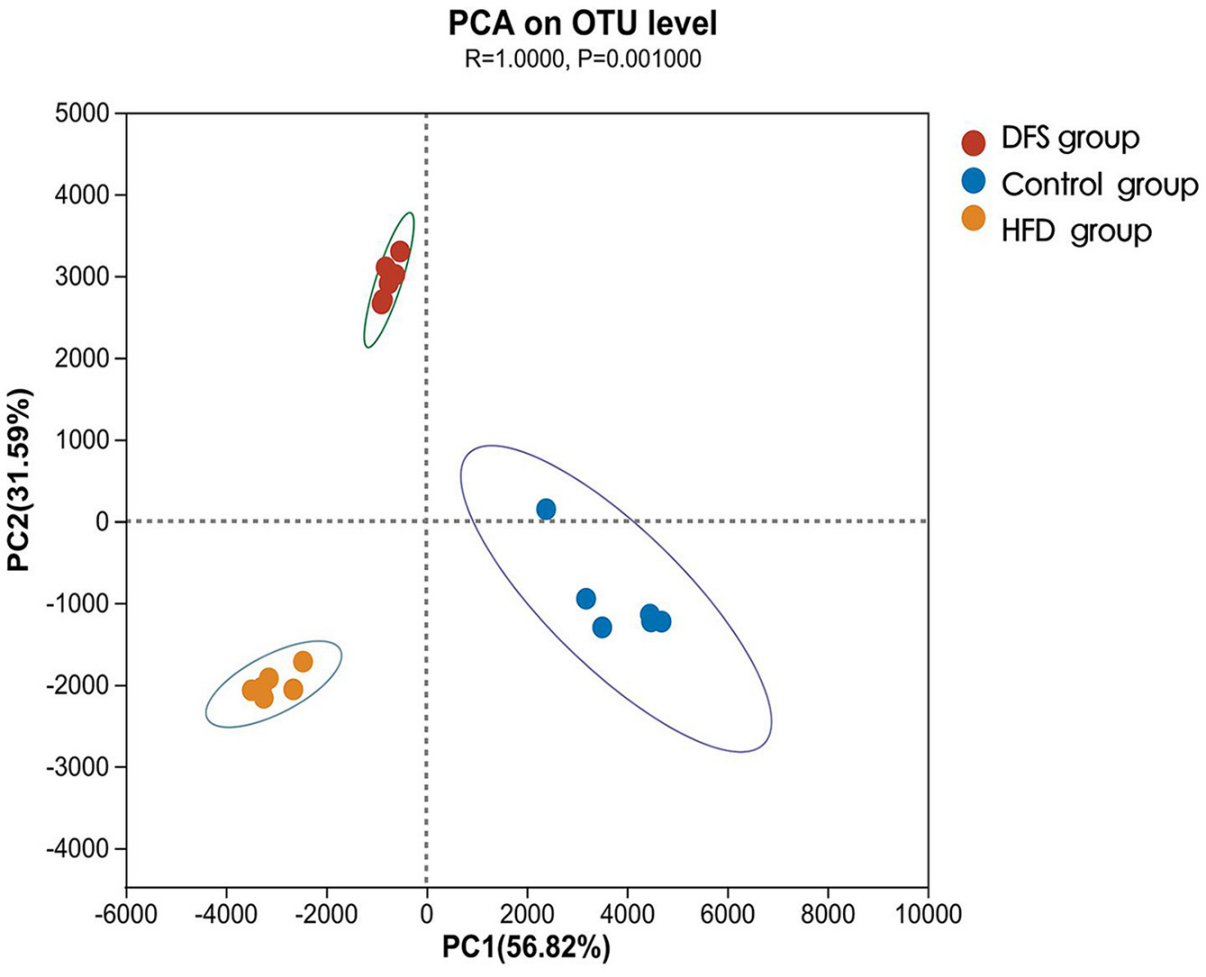
PCA of rat gut flora. DFS: Dry-Fried Soybeans; HFD: High-Fat Diet; PCA: Principal Component Analysis, with all experiments repeated six times.

The dietary structure of rats significantly influences the diversity of gut microbial communities [31]. Alpha diversity measures the variety of species within a sample. The Shannon index represents microbial diversity, while Chao and Acés indices estimate the number of species. Significant differences in the distribution of gut flora were observed among the three groups of rats (Table 3). Compared to the control group, microbial diversity increased in both the DFS and HFD groups (*p* = 0.013, *p* = 0.005), although no significant difference in the Shannon index was found between the DFS and HFD groups. According to the Chao and Ace indices, fecal microbial richness was highest in the DFS group but not significantly different from the control group. However, fecal microbial richness decreased in the HFD group compared to the DFS and control groups (*p* = 0.005). Beta diversity analyses explore differences or similarities in community composition. PCA is a technique for simplifying data analysis (Fig. 3). Results indicate that high-temperature processed feed significantly increases the diversity of gut microbial communities in rats.

### Impact of High-Temperature Processed Feed on Rat Gut Bacterial Phylum-Level Abundance

To investigate the effect of high-temperature processed feed on the gut flora structure in a rat model of non-obese MAFLD, we compared the bacterial phylum-level abundance among the DFS, HFD, and control groups.

**Fig. 4:**
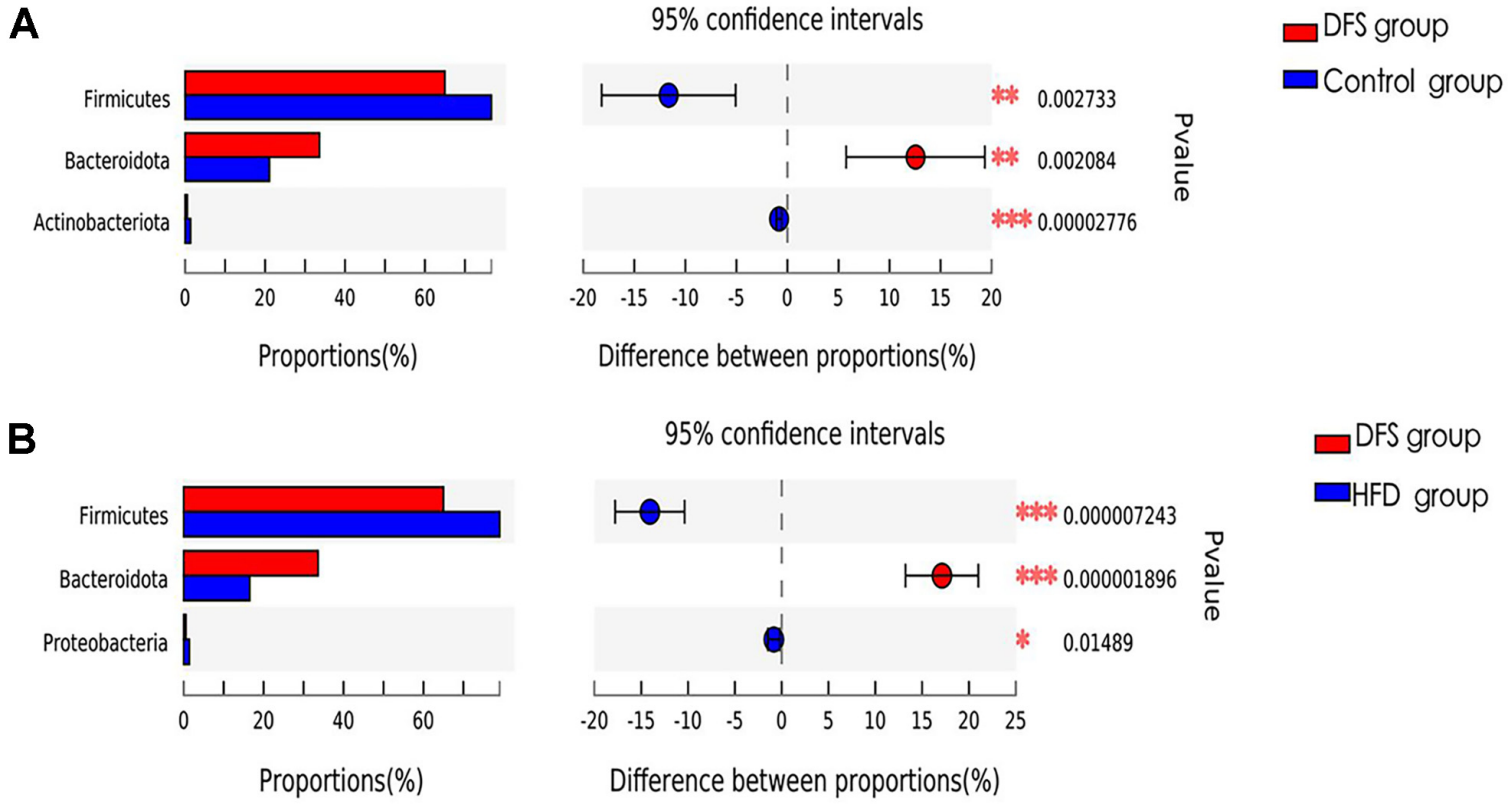
Impact of high-temperature processed feed on the average abundance of rat gut microbial phyla. (**A**) Bacterial community structure between the DFS group and the control group; (**B**) Bacterial community structure between the DFS group and the HFD group; DFS: Dry-Fried Soybeans; HFD: High-Fat Diet; *p < 0.05, **p < 0.01, ***p < 0.001, with all experiments repeated six times.

The predominant bacterial phyla in both the DFS and control groups included Bacillota, Bacteroidetes, and Actinobacteria, with the addition of Proteobacteria in both the DFS and HFD groups. Statistical analysis revealed that compared to the control group, the DFS group showed a decrease in the abundance of Bacillota and Actinobacteria and an increase in Bacteroidetes, with a slight rise in Proteobacteria. Similarly, compared to the HFD group, the DFS group exhibited a significant increase in Bacteroidetes abundance and decreased Bacillota, Actinobacteria, and Proteobacteria abundance (Fig. 4A and 4B). These differences were statistically significant (*p* < 0.05). This study demonstrates that high-temperature processed feed significantly alters the composition of rat gut flora, mainly by reducing the abundance of Bacillota, Actinobacteria, and Proteobacteria while increasing the abundance of Bacteroidetes.

### Quantitative Analysis of the Impact of High-Temperature Processed Feed on the Species Distribution of Rat Gut Flora

To better understand how high-temperature processed feed affects the distribution of genera within the gut flora, this study focused on the top 50 most abundant genera, using genus-level annotation for detailed analysis rather than species-level annotation, which would include specific species names such as Akkermansia muciniphila.

**Fig. 5:**
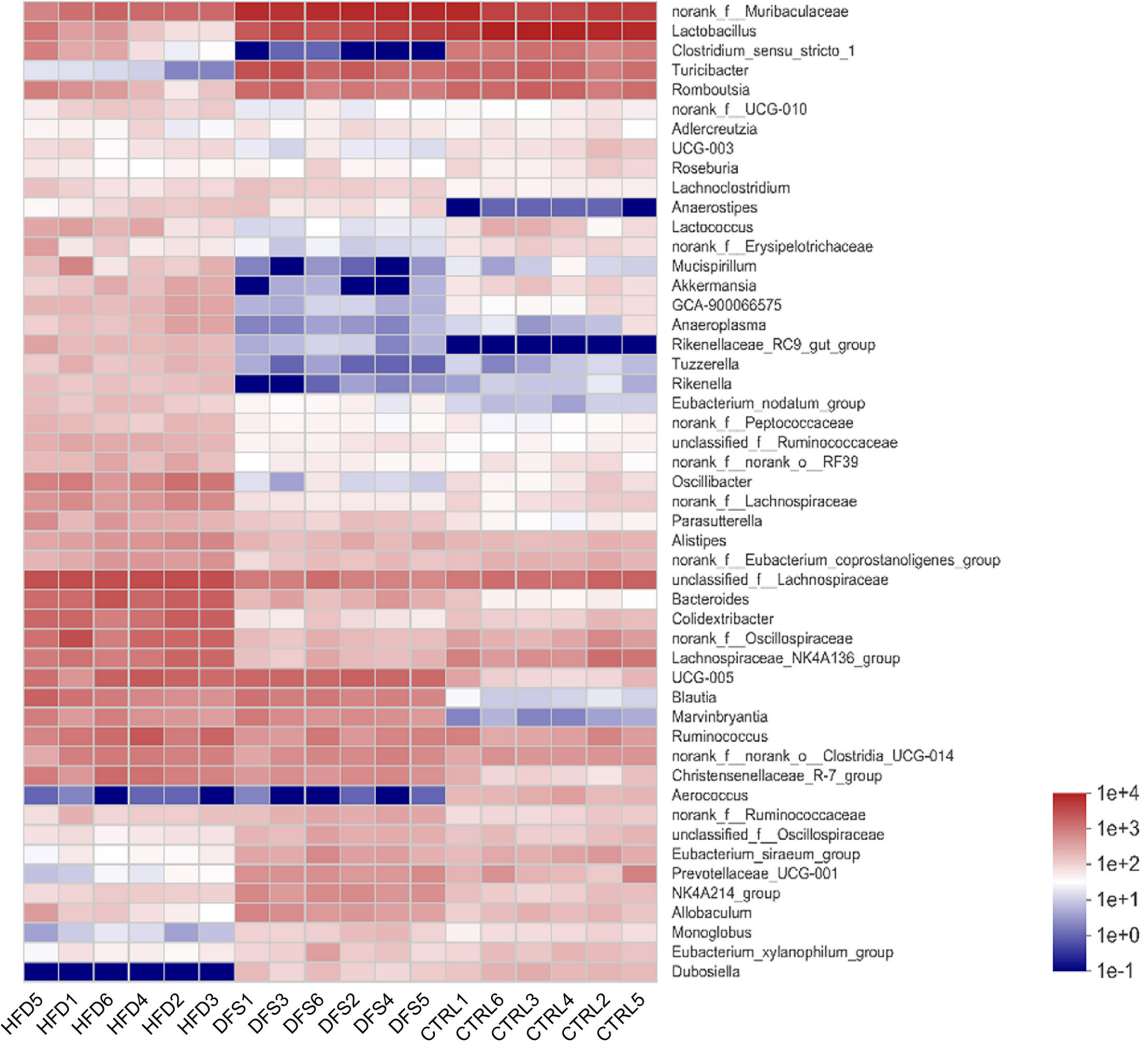
Heatmap distribution of gut flora species diversity across different feed treatment groups. The heatmap displays the top 50 genera in abundance in the HFD, DFS, and control groups. G1-6 represents samples 1 to 6 from the HFD group, c1-6 represents samples 1 to 6 from the DFS group, and n1-6 represents samples 1 to 6 from the control group. Each group was replicated six times to ensure the reliability of the results.

Heatmaps revealed variations in the distribution of gut microbial species among different dietary treatment groups in rats. Comparisons between the DFS group, HFD group, and the control group showed significant reductions in the abundance of critical genera such as *Lactococcus*, *Akkermansia*, *Mucispirillum*, *GCA-900066575*, and *Clostridium_sensu_stricto_1* in the DFS group (Fig. 5). These genera were identified as critical based on their significant changes in abundance and their known roles in gut health and disease. These findings suggest the potential impact of high-temperature processed feed on gut microbial diversity and ecological balance.

In summary, high-temperature processed feed significantly altered the species abundance of specific microbial communities in rat intestines, particularly affecting genera like *Lactococcus* and *Akkermansia*, which are associated with gut health. This may offer new microbiological perspectives for the study of the etiology of non-obese MAFLD.

### Impact of High-Temperature Processed Feed on Rat Fecal Metabolic Pathways

Dietary influences on the metabolic pathways of rat feces have been documented [46]. Differences in metabolic pathways between the control and DFS groups included arginine-ornithine metabolism, arginine-proline metabolism, protein metabolism, and absorption pathways. Compared to the control group, the DFS group exhibited an increase in fecal metabolites, such as AMC arachidonoyl, 11-eicosenoic acid, 13-hydroxy octadecanoic acid, 11-hydroxy-9-tridecenoic acid, LysoPC(0:0/18:0), linoleic acid, pyridine N-oxide glucuronide, bile acid glucuronide, LysoPC(P-18:1(9Z)), 12-oxo-20-dihydroxy leukotriene B4, LysoPE(18:0/0:0), 9-oxo nonanoic acid, 9-oxo OTrE, and LysoPC(20:1(11Z)) (Table 4).

Distinct metabolic pathways between the HFD group and DFS group included the arginine-ornithine pathway, arginine-proline pathway, tryptophan pathway, steroid biosynthesis, bile acid biosynthesis, bile secretion, cholesterol metabolism, glutathione metabolism, linoleic acid metabolism, and arachidonic acid metabolism. Relative to the HFD group, the DFS group showed an increase in fecal metabolites including 5-hydroxyindole glucuronide, docosahexaenoic acid, N-acetyl aspartate, methyl linoleate, linoleic acid, acetylcholine, bile acid glucuronide, pyridine N-oxide glucuronide, 12-oxo-20-dihydroxy leukotriene B4, cholic acid 3-O-glucuronide, and prostaglandin F1α. Increased substances in the HFD group included 5-hydroxy-L-tryptophan, DL-citrulline, tauroursodeoxycholic acid, tauroursodeoxycholate, ceramide (d18:1/9Z-18:1), and 5-trans carbaprostaglandin (Table 5). These results suggest that high-temperature processed feed increases the content of fecal metabolites in rats.

### Correlation Analysis between Gut Microbial Communities and Fecal Metabolites in Rats Fed with High-Temperature Processed Feed

**Fig. 6:**
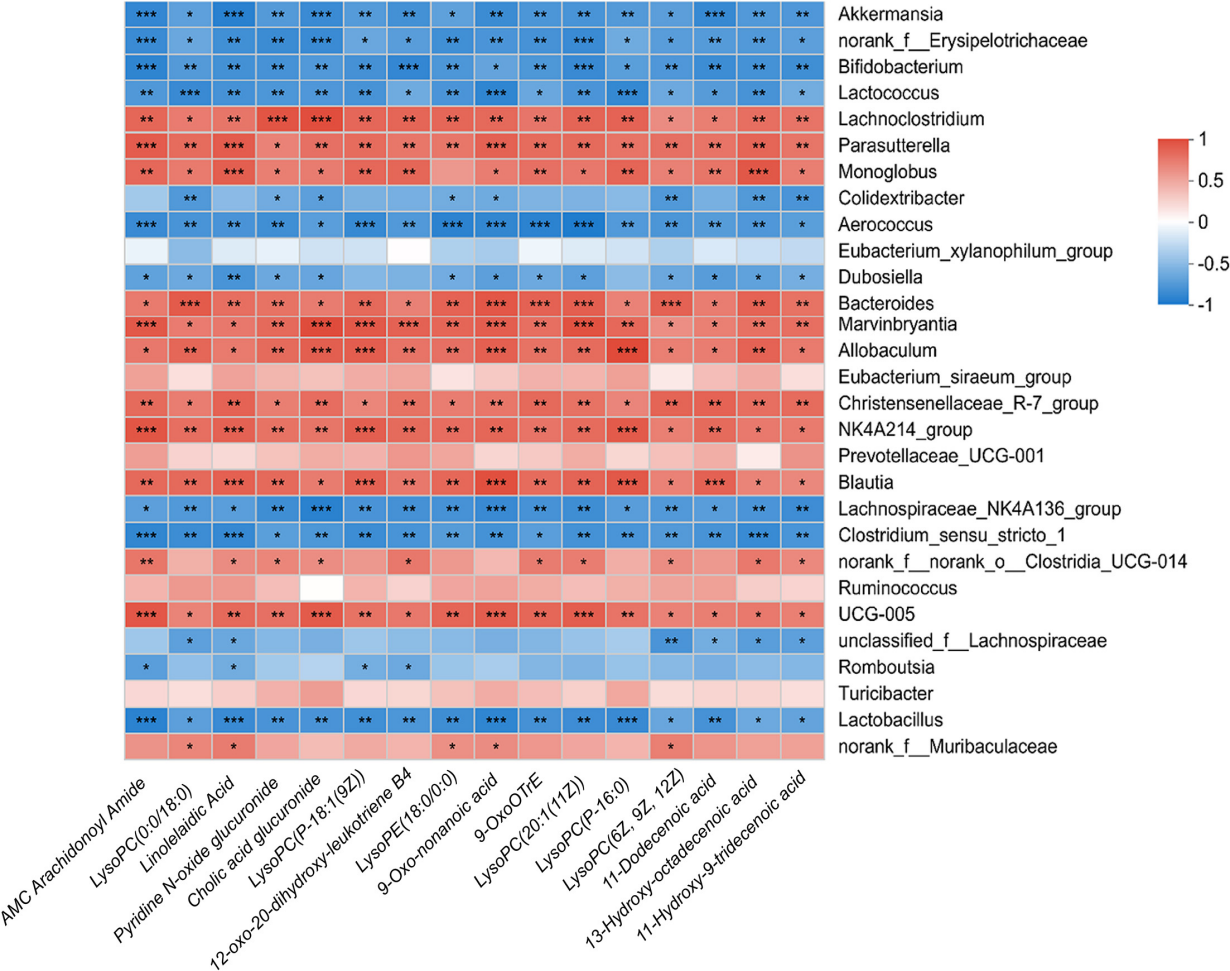
Compared to the control group, there is a correlation between gut microbial communities and fecal metabolites in the DFS group. DFS: Dry-Fried Soybeans.

Investigating the correlation between fecal metabolites and gut microbial communities in rats, we noted a close relationship between microbial community composition changes and specific metabolite levels. Previous research has highlighted the interaction between gut microbial communities and host metabolites, significantly affecting the host's health and disease states [47]. Compared to the control group, an increase in the abundance of microbial communities such as *Lachnoclostridium*, *Parasutterella*, *Monoglobus*, *Bacteroides*, *Marvinbryantia*, *Allobaculum*, *NK4A214_group*, and *Blautia* in the DFS group was positively correlated with elevated metabolites (Fig. 6). This suggests that high-temperature processed feed may influence the gut metabolic environment by altering the abundance of these microbial communities.

**Fig. 7:**
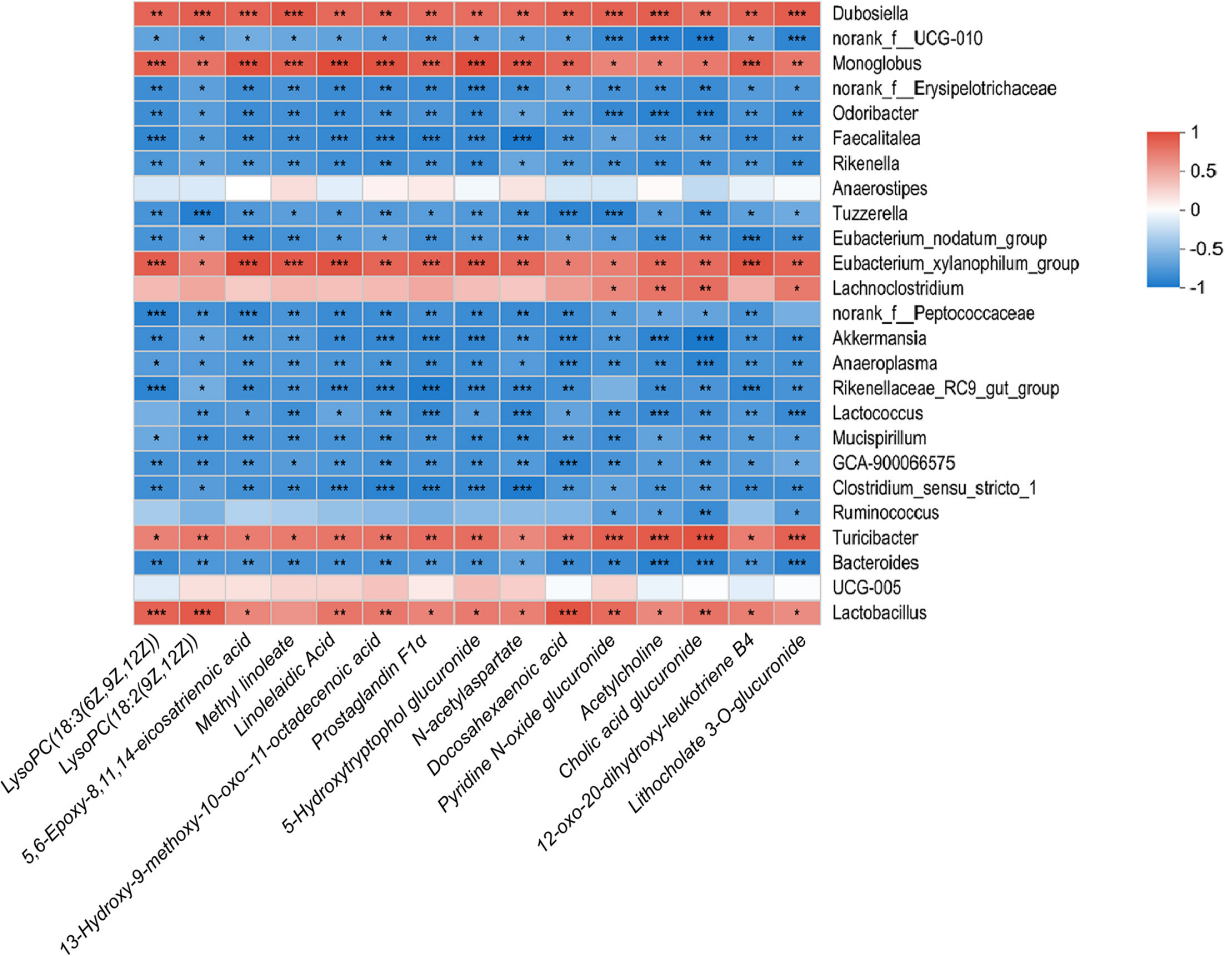
Correlation between gut microbial communities and fecal metabolites in the DFS group compared to the HFD group. DFS: Dry-Fried Soybeans, HFD: High-Fat Diet.

Conversely, the abundance of *Akkermansia*, *Bifidobacterium*, *Lactococcus*, *Aerococcus*, and *Dubosiella* was negatively correlated with elevated metabolites (Fig. 6), indicating these microbial communities may play a suppressive role in maintaining metabolic homeostasis. When compared with the HFD group, similar findings were observed, with *Lactobacillus*, *Turicibacter*, and *Eubacterium_xylanophilum*_group positively correlated with increased metabolites in the DFS group, while *Bacteroides*, *Ruminococcus*, and *Clostridium_sensu_stricto_1* showed a negative correlation (Fig. 7). These findings emphasize the complex interactions between microbial communities and metabolites, suggesting that high-temperature processed feed may affect the gut microenvironment and host health by modulating these interactions. Notably, the negative correlation with *Akkermansia* may relate to its known functions in protecting the intestinal barrier and anti-inflammatory effects, while the positive correlation with *Lachnoclostridium* could reflect changes in the gut microbial communities under non-obese MAFLD conditions.

### Correlation Analysis between Gut Flora and Liver Health in Rats Fed with High-Temperature Processed Feed

Research indicates a strong correlation between rat gut flora and liver health [48]. An analysis of the correlation between gut flora, liver index, and liver fibrosis in the DFS group compared to the control group revealed that the presence of *Akkermansia*, *Clostridium_sensu_stricto_1*, and *Mucispirillum* was inversely related to liver fibrosis scores in the DFS group. Additionally, an increased abundance of *Lactococcus* in the DFS group was positively associated with the liver index (Table 6). These findings suggest that these microbial groups play a significant role in protecting liver structure and function. Comparative analysis between the DFS and HFD groups showed a negative correlation between *Mucispirillum* and fibrosis scores in the DFS group. Within the DFS group, *Lactococcus*, *Akkermansia*, and *GCA-900066575* positively correlated with the liver index, whereas *Dubosiella*, *Lactobacillus*, and *Turicibacter* showed a negative correlation (Table 7), indicating the relationship between gut flora and liver health varies and is complex under different dietary influences.

This study demonstrates the impact of high-temperature processed feed on the composition of rat gut flora and its correlation with liver fibrosis and the liver index. The negative correlation observed with *Akkermansia*, *Clostridium_sensu_stricto_1*, and *Mucispirillum* may highlight their potential protective role in maintaining liver health. Conversely, the positive association with *Lactococcus* suggests its potential influence on changes in the liver index.

## Discussion

In our study, we selected the HFD group as a positive control because a HFD is a standard model for NAFLD. Previous findings indicated that rats on a HFD exhibited bullous steatosis, whereas NAFLD induced by high-temperature processed food showed vesicular steatosis [49-51], suggesting distinct pathogeneses. Fecal observations at week 12 revealed unformed, sticky stools in the DFS group, whereas stools from the control and HFD groups were well-formed. This underscores the pivotal role of gut flora changes in stool consistency variations. Extensive evidence supports the gut bacteria as an "energy metabolism second center" in the body [52, 53-55], with gut flora playing critical roles in nutrient digestion and absorption, beneficial nutrient production, defense against pathogenic bacteria, and immune regulation [56, 57].

The intestine and liver are interconnected through the portal vein circulation, the hepatoenteral lymphatic system, and the bile circulation of the biliary system [17, 58]. The integrity of the intestinal structure and function is vital for nutrient absorption and the synthesis and metabolism of substances within the body [59, 60]. When the intestinal barrier is compromised, increased intestinal wall permeability leads to bacterial translocation [61, 62]. This translocation allows gut bacteria, bacterial metabolites, and related inflammatory factors to enter the liver and intestines through the portal vein [63, 64]. Damage to the intestinal mucosal barrier increases intestinal permeability, and dysbiosis can significantly influx these bacteria and their products into the liver via the portal vein [65, 66]. The liver then experiences an influx of chemokines and inflammatory cytokines triggered by the nonspecific immune system, leading to or exacerbating liver inflammation and potentially progressing to cirrhosis and liver cancer [29, 67-70].

In our study, compared to the HFD and control groups, the DFS group showed a decrease in Bacillota, Actinobacteria, and Proteobacteria phyla while Bacteroidetes increased. Literature reports that the ratio of Bacillota to Bacteroidetes is elevated in obese MAFLD but reduced in non-obese MAFLD. Our findings align with various domestic and international studies [71-74]. The DFS group exhibited a reduction in *Akkermansia*, *Mucispirillum*, *Clostridium_sensu_stricto_1*, and lactic acid bacteria compared to the HFD and control groups. *Akkermansia* and *Mucispirillum*, mucosal-residing bacteria forming a microbial film, inherently protect the host by inhibiting contact between pathogens and the host mucosa. *Akkermansia*, a standard component of human gut flora, has been inversely correlated with low-grade inflammation [75]. It not only maintains the integrity of intestinal epithelial cells and the mucus layer but also exhibits anti-inflammatory effects through regulatory T cells, the endocannabinoid system, and non-classical Toll-like receptors, playing a crucial role in the low-grade chronic inflammation observed in the DFS group.

Compared to the other groups, the DFS group showed increased levels of 12-oxo-20-dihydroxy leukotriene B4 and pyridine N-oxide glucuronide. Correlation analyses indicated a positive relationship between these metabolites and *Monoglobus* and a negative correlation with *Akkermansia*, *Mucispirillum*, lactic acid bacteria, and *Clostridium_sensu_stricto_1*. The increase in pyridine N-oxide glucuronide levels in the DFS group showed a strong positive correlation with *Lachnoclostridium*, and in comparisons between the DFS and HFD groups, a strong positive correlation with *Turicibacter* and *Dubosiella*. Pyridine N-oxide glucuronide, a product of N-oxidation and oxidative stress, has been associated with decreased *Dubosiella* in the HFD group [76]. *Dubosiella* can ameliorate HFD-induced abnormalities and reduce LDL and TG in the serum and liver of obese mice [76, 77]. In this study, no significant differences were observed in body weight and lipid levels between the DFS group and the control group. However, compared to the HFD group, the DFS group showed significant reductions in lipid levels and body weight, with hepatic fat degeneration manifesting as vesicular steatosis. *Dubosiella* may play a critical role in this context.

Compared to the DFS and control groups, the HFD group exhibited increased *Ruminococcus* and *Parasutterella*, with a decrease in *Dubosiella* and *Monoglobus*. Studies have shown that *Ruminococcus* abundance is higher in patients with non-alcoholic steatohepatitis than those with simple steatosis [78-80]. Moreover, levels of taurine-conjugated bile acids, such as tauroursodeoxycholic acid, were increased in the HFD group compared to the DFS and control groups. *Parasutterella* has been associated with obesity and type 2 diabetes in humans [81, 82] and implicated in cholesterol metabolism [83, 84]. Thus, unlike the DFS group, a HFD induces changes in rat bacterial metabolites, including an increase in bile-related substances, potentially driving the progression of MAFLD.

Lactobacilli and streptococcaceae were most abundant in the control group, with their numbers reduced in both the DFS and HFD groups, particularly in the HFD group. The DFS group showed a lower liver index than both the HFD and control groups (*p* < 0.05) and a significantly increased fibrosis area (*p* < 0.01, *p*<0.05). Correlation analysis between gut flora and liver fibrosis and liver index revealed that *Lactobacillus*, *GCA-900066575*, *Bifidobacterium*, *Lactococcus*, *Dubosiella*, *Clostridium_sensu_stricto_1*, *Akkermansia*, and *Mucispirillum* were negatively correlated with fibrosis scores in the DFS group. Conversely, *Blautia*, *Marvinbryantia*, and Bacteroidetes were positively correlated. The DFS group's *Mucispirillum* showed a negative correlation with fibrosis scores, whereas *Ruminococcus*, *GCA-900066575*, *Lactobacillus*, and *Akkermansia* showed a positive correlation with the liver index in the DFS group. The increased liver index in the HFD group negatively correlated with the abundance of *Dubosiella*, *Lactobacillus*, and *Turicibacter* in the intestine.

Therefore, *Akkermansia* and *Mucispirillum* play crucial roles in the occurrence of liver fibrosis in the DFS group. The reduction of gut mucosal barrier-protective bacteria like *Akkermansia* and *Mucispirillum* in the DFS group's feces indicates that the destruction of the intestinal mucosal barrier allows harmful substances to enter the liver via the gut-liver axis, exacerbating fibrosis in the DFS group more than in the HFD and control groups.

The etiology of non-obese MAFLD remains unclear. However, the role of high-temperature processed food in the pathogenesis of MAFLD has been overlooked. We discovered an increase in oxidative stress-related substances in the feces of the DFS group and a lack of protective bacteria for the intestinal mucosal barrier, such as *Akkermansia* and *Mucispirillum*. Moreover, we found a connection between *Akkermansia*, *Mucispirillum*, and liver fibrosis in the DFS group. Thus, our research may offer new directions for understanding and treating MAFLD.

This study delves into the effects of high-temperature processed feed on a rat model of non-obese MAFLD, mainly focusing on microbial communities and liver health, thus offering more profound insights into the pathophysiology of non-obese MAFLD. Compared to the HFD group, rats fed with high-temperature processed feed (the DFS group) exhibited a lower liver index and increased liver fibrosis, suggesting the potential role of gut flora in regulating liver health. Moreover, the study highlights the protective roles of *Akkermansia* and *Mucispirillum* in maintaining intestinal integrity and resisting liver disease progression, offering new perspectives for developing microbial communities-based therapeutic strategies. Unlike previous studies, our research underscores the importance of the gut-liver axis in non-obese MAFLD and identifies key microbial members promoting liver health, laying a foundation for future clinical applications.

However, this study's limitations must be considered. Primarily, the use of an animal model and the lack of human studies limit the direct clinical applicability of our findings. Additionally, the high-temperature processed feed used in experiments differs significantly from standard human diets, potentially affecting the generalizability of the results. While we observed positive correlations between *Akkermansia*, *Mucispirillum*, and liver health, the precise mechanisms remain unclear, necessitating further research to elucidate how these microbes affect liver pathology.

Future studies should address these limitations. They conduct human clinical trials to verify the effects and safety of high-temperature processed feed and specific gut flora in treating non-obese MAFLD. This would confirm findings from animal models and assess the practical application potential in humans. Moreover, exploring the specific mechanisms of *Akkermansia* and *Mucispirillum*, especially their interactions with the host's metabolic pathways, could reveal the molecular basis of their liver-protective effects. Investigating other gut microbes and their metabolites with potential protective effects against non-obese MAFLD could broaden the scope for developing novel microbial communities-based therapies. In summary, while this study makes strides in unveiling the gut-liver axis mechanisms in non-obese MAFLD, realizing its scientific and clinical value further depends on more in-depth and extensive future research.

## Conclusion

**Fig. 8:**
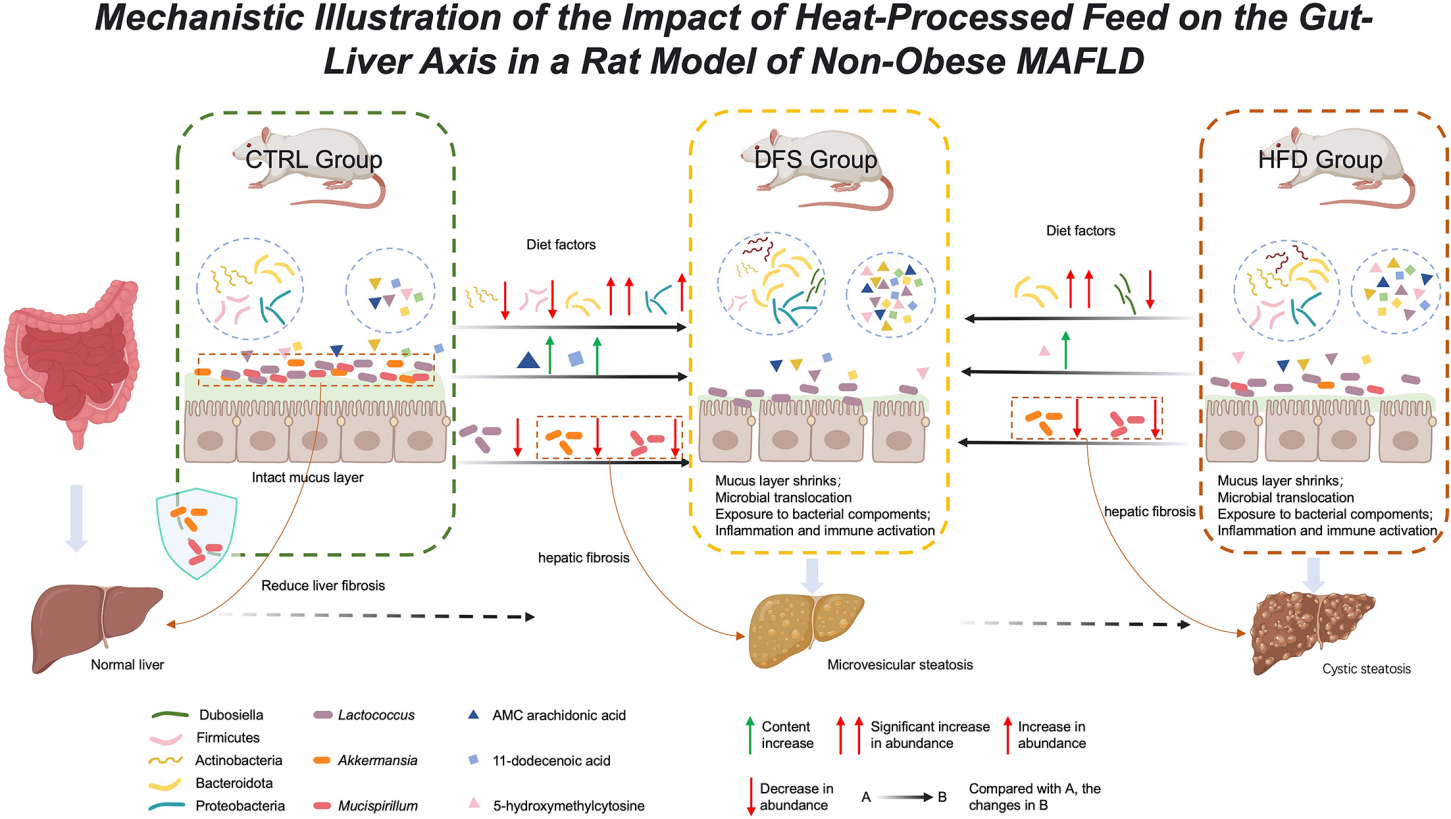
Mechanistic illustration of the impact of heat-processed feed on the gut-liver axis in a rat model of non-obese MAFLD.

This study unveils the potential impacts of high-temperature processed feed on non-obese MAFLD, highlighting its strong association with gut flora. It is the first to report that high-temperature processed feed can contribute to the development of non-obese MAFLD, characterized by varying types of hepatic fat degeneration. The decrease of *Akkermansia* and *Mucispirillum* in the DFS group was found to be associated with liver fibrosis. These findings offer new insights into the mechanisms of MAFLD and provide clues for future disease treatment strategies, especially interventions targeting gut flora (Fig. 8).

## Figures and Tables

**Table 1:** Comparison of liver index and fibrosis grading among three groups at week 12.

Group	Number	Fibrosis (score)				
		0	1	2	3	4
Control group	6	6	0	0	0	0
DFS group	6	0	0	4	2	0
HFD group	6	0	1	5	0	0

DFS: Dry-fried soybeans; HFD: high-fat diet. 0 (no fibrosis), 1 (mild fibrosis), 2 (moderate fibrosis), 3 (severe fibrosis), and 4 (cirrhosis)

**Table 2:** Comparison of Intestinal Permeability and Integrity among the Three Groups at Week 12.

Group	Sample size	Serum zonulin level (ng/ml)	Lactulose/mannitol ratio
Control group	6	30.5 ± 2.3	0.04 ± 0.01
DFS group	6	45.7 ± 3.1*	0.07 ± 0.02*
HFD group	6	60.2 ± 4.5**	0.11 ± 0.03**

*p value for 0.05, ** *p* value for 0.01

**Table 3:** Statistical significance of comparison of Alpha diversity between the three groups.

	Shannon index	Shannon	Chao index	Chao	Ace index	Ace
Control group - DFS group	3.50 vs 3.80*	0.013*	85 vs 87	0.936	80 vs 83	0.378
Control group - HFD group	3.50 vs 2.90**	0.005**	85 vs 70^##^	0.005^##^	80 vs 71^##^	0.005^##^
DFS group - HFD group	3.80 vs 2.90	0.230	87 vs 70^##^	0.005^##^	83 vs 71^##^	0.005^##^

*p value for 0.05, ** *p* value for 0.01 ##*p* value for 0.01

**Table 4:** Increased differential metabolites in the DFS group compared with Control group.

Metabolite	VIP_PLS-DA	FC(DFS/Control)	*p*-value
11-Dodecenoic acid	1.082	1.065	<0.001
AMC Arachidonoyl Amide	1.769	1.210	<0.001
LysoPC(0:0/18:0)	1.341	1.091	<0.001
Linoelaidic Acid	1.178	1.070	<0.001
13-hydroxyoctadecanoic acid	1.045	1.080	<0.001
Pyridine N-oxide glucuronide	2.616	1.727	<0.001
Cholic acid glucuronide	1.339	1.115	<0.001
LysoPC(P-18:1(9Z))	1.121	1.095	<0.001
12-oxo-20-dihydroxy-leukotriene B4	1.340	1.152	<0.001
LysoPE(18:0/0:0)	1.551	1.154	<0.001
9-Oxo-nonanoic acid	1.002	1.075	<0.001
9-OxoOTrE	1.008	1.074	<0.001
LysoPC(20:1(11Z))	1.234	1.120	<0.001
LysoPC(P-16:0)	1.454	1.157	<0.001
LysoPC(18:3(6Z,9Z,12Z))	1.561	1.209	<0.001
11-Hydroxy-9-tridecenoic acid	1.344	1.220	<0.001

FC: fold change

**Table 5:** Increased differential metabolites in the DFS group compared with HFD group.

Metabolite	VIP_PLS-DA	FC(HFD/DFS)	*p-*value
Methyl linoleate	1.961	0.642	<0.001
Linoelaidic Acid	1.302	0.852	<0.001
13-Hydroxy-9-methoxy-10-oxo-11-octadecenoic acid	1.208	0.826	<0.001
Prostaglandin F1a	1.323	0.818	<0.001
Pyridine N-oxide glucuronide	2.163	0.465	<0.001
12-oxo-20-dihydroxy-leukotriene B4	1.154	0.813	<0.001
LysoPC(18:2(9Z,12Z))	1.472	0.776	<0.001
N-acetylaspartate	1.112	0.850	<0.001
Lithocholate 3-O-glucuronide	1.378	0.770	<0.001
Docosahexaenoic acid	2.132	0.432	<0.001
LysoPC(18:3(6Z,9Z,12Z))	2.085	0.419	<0.001
5,6-Epoxy-8,11,14- eicosatrienoic acid	1.377	0.808	<0.001
5-Hydroxytryptophol glucuronide	1.094	0.878	<0.001
Acetylcholine	1.086	0.884	<0.001
Cholic acid glucuronide	1.276	0.824	<0.001

FC: fold change

**Table 6:** Correlation between liver fibrosis, liver index and gut microbiota between DFS and control groups.

	Fibrosis		Liver index	
	R value	P value	R value	P value
Lactobacillus	-0.881	<0.001	0.400	0.198
Clostridium_sensu_stricto_1	-0.927	<0.001	0.512	0.089
Blautia	0.948	<0.001	-0.365	0.243
Marvinbryantia	0.859	<0.001	-0.440	0.153
Bacteroides	0.754	0.005	-0.280	0.377
Dubosiella	-0.632	0.027	0.401	0.196
Lactococcus	-0.659	0.020	0.584	0.046
Bifidobacterium	-0.876	<0.001	0.475	0.118
Akkermansia	-0.847	0.001	0.403	0.194
GCA-900066575	-0.792	0.002	0.189	0.557
Mucispirillum	-0.631	0.028	0.114	0.723

**Table 7:** Correlation between liver fibrosis, liver index and gut microbiota between DFS and HFD groups.

	Fibrosis		Liver index	
	R value	P value	R value	P value
Lactobacillus	0.409	0.187	-0.912	<0.001
Turicibacter	0.536	0.073	-0.863	<0.001
Ruminococcus	-0.183	0.569	0.723	0.008
Clostridium_sensu_stricto_1	-0.295	0.351	0.459	0.133
GCA-900066575	-0.427	0.166	0.929	<0.001
Mucispirillum	-0.719	0.008	0.534	0.074
Lactococcus	-0.575	0.050	0.717	0.009
Akkermansia	-0.322	0.307	0.845	0.001
Dubosiella	0.513	0.088	-0.869	<0.001
